# Beyond degradation tags: How FAT10 and ubiquitin shape substrate energy landscapes

**DOI:** 10.1042/EBC20253034

**Published:** 2025-11-06

**Authors:** Aravind Ravichandran, Adarshan Sivakumar, Ranabir Das

**Affiliations:** National Centre for Biological Sciences, TIFR, Bangalore, India

**Keywords:** protein–protein interactions, thermodynamics, ubiquitin–proteasome system

## Abstract

Protein degradation via the proteasome is a fundamental process for maintaining proteostasis. The post-translational modification of substrate proteins by ubiquitin and the ubiquitin-like modifier FAT10 targets them for proteasomal degradation. While ubiquitin and FAT10 have traditionally been perceived as passive signals for proteasomal targeting, emerging evidence indicates that they actively influence both the thermodynamic and conformational landscapes of their respective substrates. In this review, we explore recent mechanistic insights into how the modification site and the intrinsic characteristics of the modifier dictate substrate stability. Ubiquitin destabilizes proteins in a site-specific manner through entropic restriction or enthalpic disruption, thereby modulating degradation efficiency. It is noteworthy that well-folded ubiquitin substrates require unfoldases such as p97/valosin-containing protein for successful degradation. Conversely, FAT10 acts as a significant destabilizer across various substrates due to its inherent low thermodynamic stability and flexible structure, thereby facilitating rapid degradation independent of unfoldases. These findings redefine post-translational tagging as an active regulator of protein fate and propose novel strategies for manipulating protein turnover within disease contexts.

## Introduction

Protein turnover is a fundamental biological process for cellular homeostasis and survival under stress conditions. The tightly regulated balance between protein synthesis and degradation enables cells to maintain the integrity of the proteome and adapt efficiently to stress. In eukaryotic cells, protein degradation primarily occurs through two major pathways: the autophagy-mediated system [1,2] and the proteasome-mediated system [3,4]. Autophagy typically manages the bulk degradation of long-lived proteins, protein aggregates, and organelles, and the proteasome selectively and timely degrades short-lived, regulatory, and misfolded proteins. Among these, the proteasome pathway plays a dominant role, accounting for the degradation of nearly 80% of intracellular proteins under normal physiological conditions [5].

The discovery of ubiquitin transformed our understanding of protein degradation by demonstrating that substrate proteins can be post-translationally modified with another protein, which tags them for degradation by the macromolecular proteasome machinery. In addition to its role in proteasomal degradation, ubiquitination also regulates cellular processes such as DNA repair, endocytosis, autophagy, and immune signaling [6,7]. Different ubiquitin chain topologies, like Lys48-linked [8,9] chains that signal degradation and Lys63-linked chains that participate in signaling pathways [10,11], illustrate the complexity and versatility of ubiquitin-mediated regulation. Given its broad influence, dysregulation of ubiquitin signaling has been linked to numerous diseases, such as cancer and neurodegenerative disorders, underscoring its importance in cellular function and stability [12–15].

Reprogramming the cellular environment is essential during infection to activate the immune response and for antigen presentation. Several genes essential for these processes reside within the major histocompatibility complex (MHC) locus on chromosome 6, particularly in its Class I region. The ubiquitin-like protein FAT10 (Ubiquitin D (UBD)/Human Leukocyte Antigen (HLA)-F adjacent transcript 10) is also encoded within this MHC Class I region [16,17]. Furthermore, FAT10 expression is highly inducible under inflammatory conditions, suggesting its crucial role in immune responses and pathogen defense [18]. Apart from ubiquitin, FAT10 is the only ubiquitin-like protein that tags protein for direct proteasomal degradation [19,20]. Unlike ubiquitin, which primarily functions as a reusable post-translational modifier, FAT10 is degraded with substrate at the proteasome [21,22]. This property allows FAT10 to serve as a swift degradation signal without requiring poly-chain elongation. Emerging evidence has shown that FAT10ylation may also influence their thermodynamic stability and folding dynamics before proteolysis [23,24]. Interestingly, similar effects have also been observed in substrates of ubiquitin [25–27]. This review critically examines the mechanistic aspects of FAT10 and ubiquitin’s impact on protein energy landscape, postulating that, apart from functioning as mere barcodes for the proteasome, these molecules modulate substrate energetics to ensure efficient degradation.

## Proteasome: Eukaryotic protein degradation machinery

**Figure 1:**
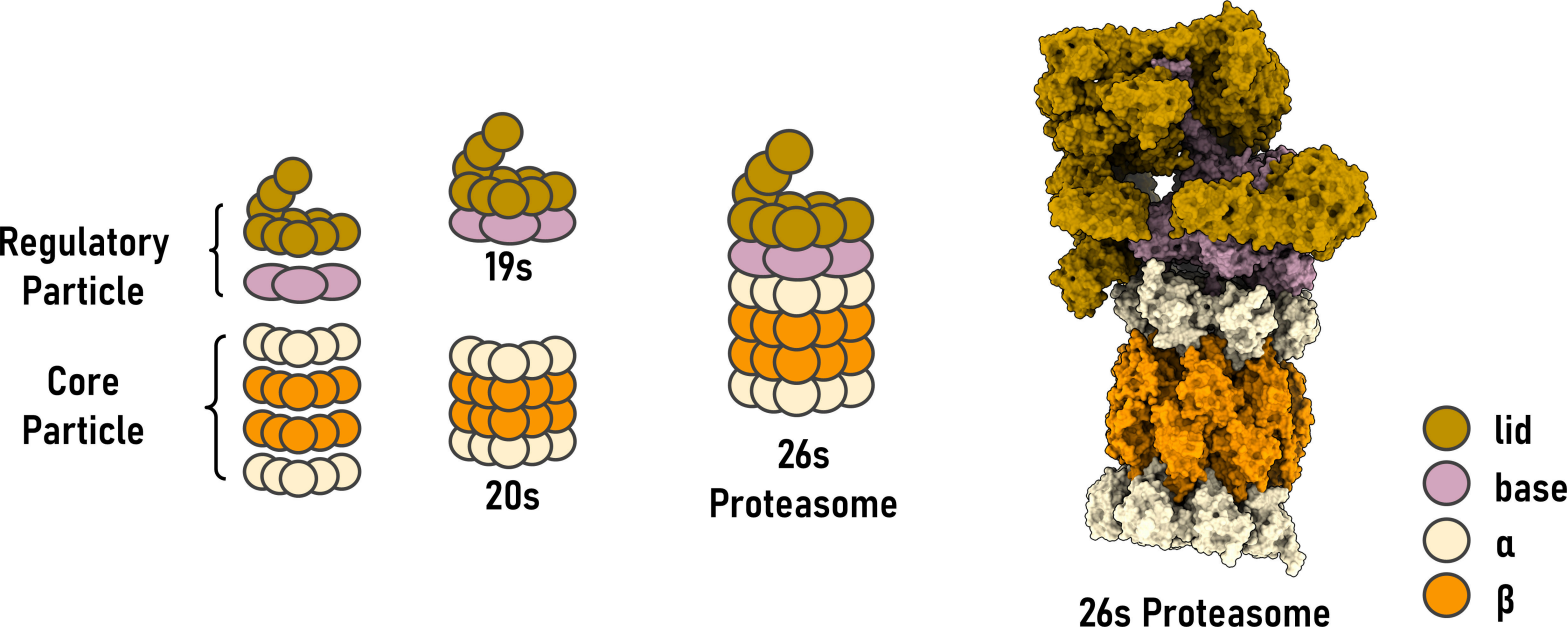
Structural organization of the 26S proteasome complexes. Schematic representations (left) and surface-rendered structure (right) illustrate the modular architecture of the eukaryotic 26S proteasome. The 19S regulatory particle (RP) comprises a lid (gold) and base (purple), while the 20S core particle (CP) consists of outer α-rings (beige) and inner β-rings (orange). The assembled 26S proteasome includes the 20S CP capped by a 19S RPs.

Proteasome is a large, multi-subunit protease complex responsible for degrading intracellular proteins in virtually all eukaryotic cells [28,29]. This 700-kDa complex, known as the 20S proteasome (Figure 1), has a cylindrical, barrel-like architecture composed of 28 subunits arranged in four stacked heptameric rings. The two outer rings consist of structurally distinct but catalytically inactive α-subunits (α1–α7), which function as a gate for substrate entry. The two inner rings are formed by β-subunits (β1–β7) and house the proteolytic chamber.

The β-subunits confer three distinct proteolytic activities: chymotrypsin-like, trypsin-like, and caspase-like, which work co-operatively to cleave a broad range of peptide bonds [30–33]. The 20S proteasome can autonomously degrade proteins containing unstructured or intrinsically disordered regions without requiring ATP or any specific degradation tag [34]. Under basal conditions, the 20S complex exists in a latent, closed-gate conformation that prevents indiscriminate degradation of cellular proteins. Multiple lines of evidence suggest that disordered regions within substrate proteins can transiently interact with surface residues of the α-ring [35]. These interactions, particularly when multivalent, appear to induce asymmetric conformational rearrangements that destabilize the axial gate, ultimately permitting substrate entry into the proteolytic chamber [35–37]. Additionally, the 20S proteasome can be capped at one or both ends by regulatory complexes such as PA28 or PA200, facilitating gate opening and enhancing proteolytic activity [38]. However, despite this intrinsic capability, the 20S complex lacks substrate selectivity and is inefficient at degrading well-folded, compact proteins [35].

To confer specificity and enable the ATP-dependent degradation of folded proteins, the 20S core can associate with one or two 19S regulatory particles (RPs), forming a 26S or 30S proteasome (Figure 1), respectively [39]. These caps recognize protein degradation signals [40], unfold them [41], and translocate them into the proteolytic chamber [42]. The modular assembly enables the proteasome to function as a versatile degradation machine, capable of selective and non-selective protein turnover.

Selective protein degradation is mediated by the 26S and 30S proteasomes, which provide spatial and temporal control over proteolysis. This specificity is conferred by the 19S RP, an ~1-MDa multi-subunit complex that caps one or both ends of the 20S proteasome core [43,44] (Figure 1). The 19S RP is organized into two major functional modules: the base and the lid [37,45–47]. The base complex comprises six ATPase subunits (Rpt1–Rpt6) arranged in a hexameric ring and four non-ATPase subunits (Rpn1, Rpn2, Rpn10, and Rpn13). The ATPases are a molecular motor that utilizes ATP hydrolysis to drive substrate unfolding and translocation [48]. The ATPase pore undergoes conformational changes coupled to ATP binding and hydrolysis, effectively threading the substrate through the central channel of the 20S core. This ATP-driven action is crucial as the 20S catalytic core degrades only unfolded or loosely structured polypeptides; tightly folded substrates must first be denatured before degradation can proceed [49]. The non-ATPase components of the base function primarily in substrate recognition and positioning. Specifically, Rpn1, Rpn10, and Rpn13 act as intrinsic ubiquitin receptors [50]. The lid complex of the 19S RP comprises at least nine non-ATPase subunits: Rpn3, Rpn5–Rpn9, Rpn11, Rpn12, and Rpn15, which are positioned atop the base and play an important regulatory role [51].

Post-translational modifications can serve two general purposes: (i) directly altering the physicochemical properties of a substrate, or (ii) acting as recognition signals for downstream effector proteins. Modifications such as phosphorylation and glycosylation can fulfill both roles [52–55]. In contrast, ubiquitination and FAT10ylation have historically been viewed primarily as recognition tags that guide proteins to the proteasome. However, for efficient degradation, the signal tag must not only direct substrates to the proteasome but also increase the dwell time of the substrate–proteasome interaction. A longer residence time at the proteasome enhances the likelihood of productive engagement, unfolding, and translocation, particularly for complex or highly structured substrates [56]. One key strategy to prolong this interaction is via the ubiquitin- or FAT10-binding shuttle factors. These adaptor proteins, such as Rad23, Dsk2, and Ddi1 for ubiquitin [57,58], and NUB1L for FAT10 [59–61], bind both the degradation tag and the proteasome, effectively tethering the substrate to the proteasome with higher affinity than the direct tag-proteasome interaction [62–64], increasing the on-rate and reducing the off-rate. However, tagging substrates with proteasome tags and shuttling factors is insufficient to guarantee efficient proteolysis, as evident from the slow degradation of ubiquitin-tagged well-folded proteins [24].

**Figure 2:**
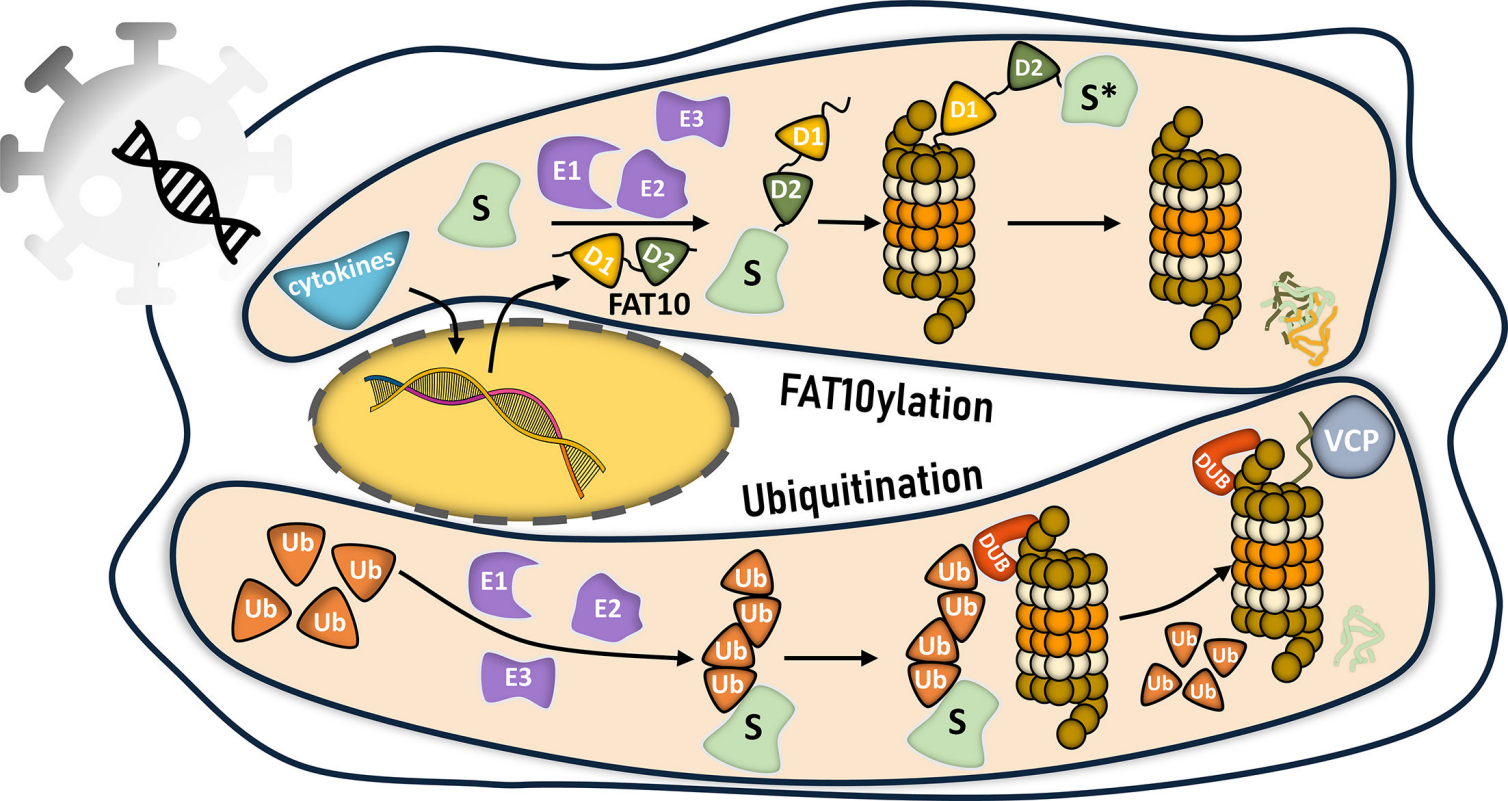
Comparison of FAT10 and ubiquitin–proteasome pathways. Ubiquitination: Multiple ubiquitin moieties are sequentially conjugated onto substrate proteins through an enzymatic cascade, forming a polyubiquitin chain. This chain is recognized by the proteasome, where ubiquitins are cleaved off prior to substrate degradation. Stable, globular substrates often require the assistance of unfoldases such as VCP/p97 for efficient degradation. FAT10ylation: In response to immune stimuli, FAT10 is expressed and conjugated to substrates via a similar enzymatic cascade. The F10–substrate complex is directly recognized by the proteasome. Both FAT10 and the substrate are degraded together in a process that does not require unfoldases activity.

A key determinant of successful engagement with the proteasome is the presence of an unstructured or loosely folded region in the substrate protein [65]. These regions act as an initiation site for the proteasome’s hexameric AAA+ ATPase motor to pull and unfold the polypeptide. The proteasome co-ordinates with external unfoldases for several substrates, such as the ATP-dependent chaperone valosin-containing protein (VCP)/p97, to overcome this barrier [66] (Figure 2). VCP is critical in extracting ubiquitinated proteins from membranes and disassembling protein complexes, thereby delivering substrates in an unfolded conformation to the proteasome [67]. The relation between substrate disorder and proteasome degradation has been reviewed elsewhere [68]. The observation that proteasome-mediated degradation can still proceed in the absence of VCP activity in many instances suggests the existence of alternative mechanisms for generating or exposing these crucial initiation regions within substrate proteins. Recent studies have highlighted the role of proteasome targeting tags toward this process, which we discuss in detail below.

Ubiquitin is a highly conserved 76-amino-acid protein that serves as a post-translational modifier, regulating various cellular processes primarily through covalent attachment to substrate proteins via isopeptide bond or through protein–protein interaction. Structurally, ubiquitin adopts a compact, globular fold called the β-grasp fold, consisting of five β-strands forming a β-sheet, a single ɑ-helix, and several loop regions [69]. This structural arrangement provides a stable yet flexible scaffold, allowing ubiquitin to interact with many substrate proteins and regulatory enzymes. Ubiquitin’s C-terminal Gly-Gly motif facilitates covalent conjugation to lysine residues on target proteins via an isopeptide bond [70]. This modification is catalyzed through a cascade of enzymatic reactions involving E1 (ubiquitin-activating enzyme), E2 (ubiquitin-conjugating enzyme), and E3 (ubiquitin ligase) enzymes [71] (Figure 2). Additionally, ubiquitin contains seven lysine residues (Lys6, Lys11, Lys27, Lys29, Lys33, Lys48, and Lys63) and Met1, each of which can serve as a linkage point for polyubiquitin chain elongation, and each linkage has distinct functional outcomes [72]. Beyond homotypic chains, ubiquitin can also form branched or mixed-linkage chains, where two or more ubiquitin moieties are simultaneously attached to multiple lysines on a single ubiquitin, resulting in complex topologies [73]. These branched polyubiquitin chains introduce an additional layer of regulatory complexity.

FAT10 is a ubiquitin-like modifier composed of two tandem domains, each adopting a β-grasp fold reminiscent of ubiquitin [22]. Despite this structural similarity, FAT10 differs significantly in function—it does not form polymeric chains. Instead, conjugation with a single FAT10 moiety directly signals proteasomal degradation. The architecture of FAT10 includes the N-terminal ubiquitin-like (UBL) domain (D1) and a C-terminal domain (D2), which are connected by a flexible linker. The C-terminal diglycine (Gly-Gly) motif of FAT10 is essential for its covalent attachment to target proteins, which is catalyzed by the FAT10-specific enzymatic cascade involving E1 (Ubiquitin-like modifier-activating enzyme 6 (UBA6)) [74,75], E2 (UBA6-specific E2 enzyme (USE1)) [76], and the E3 ligase Parkin [77]. Despite structural similarities with ubiquitin, FAT10 exhibits a lower thermodynamic stability due to its inherent flexibility, particularly due to a lack of certain key long-range electrostatic interactions in its structure [24]. This flexibility may contribute to the proteasome’s rapid recognition and degradation, distinguishing FAT10ylation from ubiquitination.

## Structural and functional overview of ubiquitin and FAT10

FAT10 has unique structural properties. Unlike ubiquitin, which can participate in diverse regulatory pathways through different chain topologies, FAT10 serves primarily as a degradation tag. Both ubiquitin and FAT10 direct substrate proteins to the proteasome, but they do so through different mechanisms: The formation of polyubiquitin chains typically serves as a strong degradation signal [78,79] (Figure 2). In contrast, mono-fat10ylation is enough for a rapid turnover of proteins by bypassing the requirement for chain elongation [20]. Interestingly, FAT10-mediated degradation proceeds independently of VCP/p97, which is otherwise crucial for the ubiquitin-dependent degradation of structured proteins [22,80–83], further emphasizing the impact of the degradation tag on the thermodynamic properties of the substrate.

## Ubiquitin as modulator of protein stability

It is plausible that appending a protein domain like ubiquitin could influence the target protein’s energetic profile and dynamic behavior. Biophysical studies have long established that domain–domain interactions within multidomain proteins can alter the stability of individual domains [84–86]. Studies reveal that the biophysical consequences of ubiquitination extend far beyond a simple degradation signal. Substantial research by several laboratories [25,27,87,88] demonstrates that ubiquitination exerts site-specific effects on substrate stability by modulating conformational energy landscapes, with profound implications for proteasomal processing. These findings revise long-standing assumptions about ubiquitin’s role as a passive tag and highlight its active role in tuning protein energetics and proteasomal engagement.

### Entropic and enthalpic modulation by ubiquitination

Computational studies showed that ubiquitin can markedly influence native state dynamics of substrates, even without specific interactions between them [87,89]. Polyubiquitination leads to decreased conformational entropy in folded regions of the substrate. This reduced entropy restricts conformational fluctuations within the native ensemble, leading to destabilization, thereby potentially accelerating degradation. Importantly, this effect was highly site-specific: the impact on stability varied significantly depending on the lysine modified, despite similar nonspecific ubiquitin–substrate interfaces. This entropic mechanism contrasts with canonical views emphasizing destabilization via interference with the unfolded state [89]. In this case, the presence of ubiquitin shifts the equilibrium by constraining the folded ensemble, directly regulating the substrate’s folding landscape.

### First experimental validation of ubiquitin-induced destabilization

**Figure 3:**
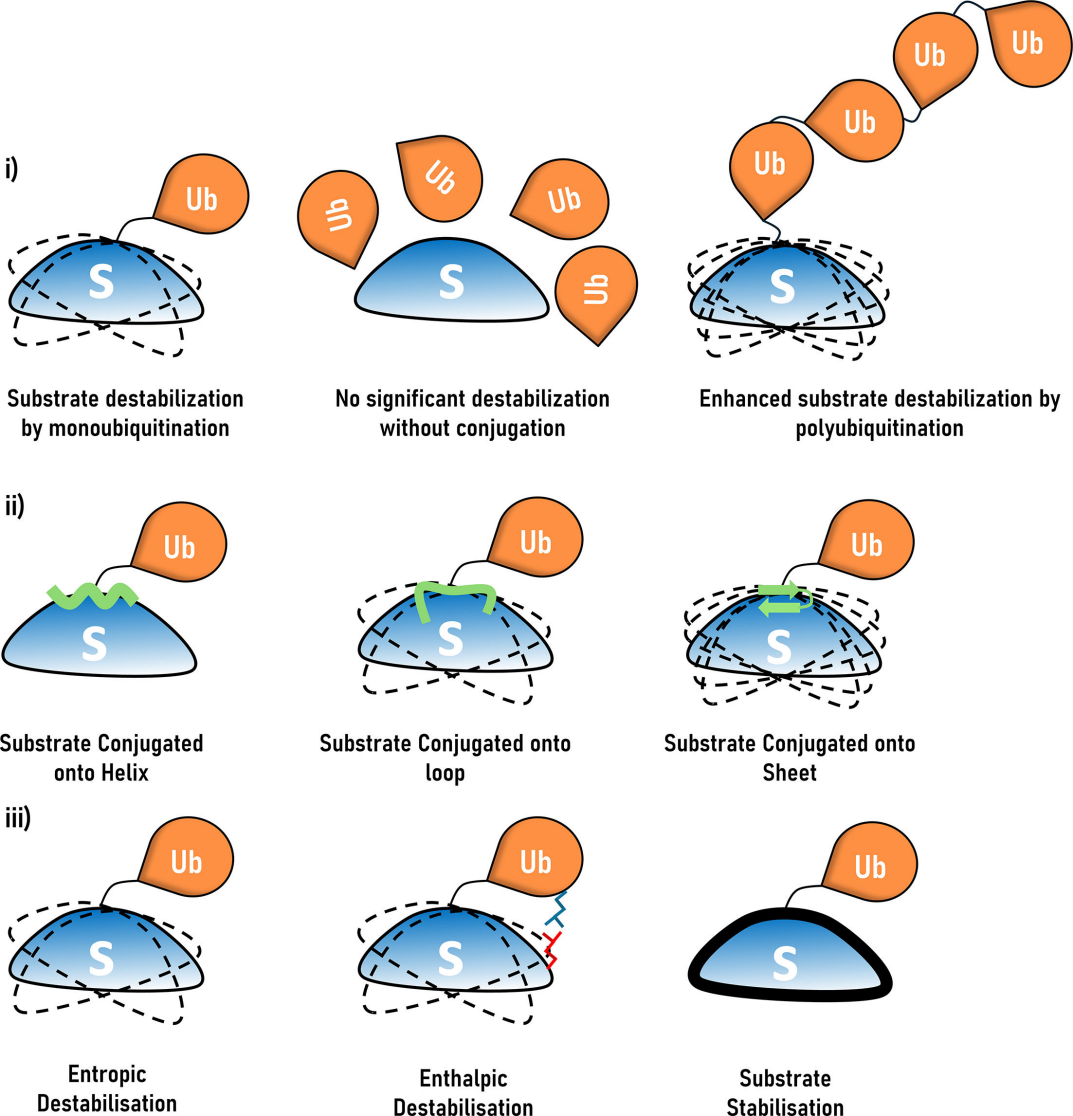
Fate of ubiquitinated substrates. Dashed outlines represent destabilization, with increased lines indicating greater destabilization. Thick outlines indicate substrate stabilization relative to the unmodified form. (**i**) Effect of monoubiquitination vs polyubiquitination: Substrates conjugated to a single ubiquitin get destabilized (left). Such destabilization effect was not observed when the substrate was mixed with ubiquitin without conjugation (middle). The extent of destabilization increased with polyubiquitination compared with monoubiquitination (right). (**ii**) Effect of secondary structure to which the ubiquitin is conjugated: Ubiquitin conjugated onto a helix showed no significant destabilization (left). Ubiquitin conjugated onto an unstructured region showed significant destabilization, while ubiquitin conjugated onto a β-sheet showed maximum destabilization. (**iii**) Types of substrate perturbation upon ubiquitin conjugation: Ubiquitination can result in distinct biophysical outcomes depending on the conjugation context. It can lead to entropic destabilization (left), often due to restriction of conformational flexibility or introduction of steric strain; enthalpic destabilization (middle), arising from disruption of native intramolecular interactions; or substrate stabilization (right), wherein ubiquitin conjugation promotes or maintains the structural integrity of the substrate.

In parallel with the computational studies, Morimoto et al. [88] provided direct experimental evidence that ubiquitination intrinsically destabilizes substrate protein folds. They showed that both N-terminal and site-specific chemical ubiquitylation led to marked decreases in thermal stability of model substrate proteins FK506-binding protein 12 (FKBP12) and Fatty acid-binding protein 4 (FABP4). These studies established that:

- Monoubiquitylation at physiological lysines reduced thermal unfolding transition temperatures by ~5–10 K (Figure 3i **left**).
- Polyubiquitylation (Ub₆) further enhanced this destabilization, suggesting a cumulative physical effect (Figure 3i **right**).
- Ubiquitination-induced destabilization was site-dependent and varied with secondary structural context: β-sheet ubiquitylation induced more substantial destabilization than α-helical or loop regions (Figure 3ii).

Notably, control experiments in this work demonstrated that physical mixing of ubiquitin with substrate proteins did not induce destabilization, affirming that a covalent linkage is essential (Figure 3i, **middle**). Furthermore, NMR relaxation data and spectral density analysis revealed increased backbone fluctuations in ubiquitylated proteins, particularly at the micro- to millisecond timescale, consistent with partial unfolding [88]. Unlike the unmodified counterpart, the Ub-modified substrate lost its refolding capacity after heat treatment, suggesting modulation of the substrate’s energy landscape.

### Site-specificity in destabilization of ubiquitination

Thermodynamic studies [25–27] with site-specifically monoubiquitinated variants of the model protein barstar revealed that the energetics of ubiquitination are highly dependent on the conjugation site. For instance, barstar ubiquitinated at Lys2 or Lys60 exhibited substantial destabilization (ΔΔG_unfolding_ of ~2.5 and ~1.9 M urea), whereas modification at Lys78 had minimal impact. These differences were not due to global structural rearrangements but reflected altered energetic landscapes—specifically, a lowered barrier to accessing partially unfolded conformations necessary for proteasomal engagement. Using an integrated model from NMR spectroscopy, hydrogen-deuterium exchange-MS, and molecular dynamics simulations, they suggested that ubiquitination does not cause large-scale structural disruption but subtly reshapes conformational fluctuations in the substrate. Sensitive ubiquitination sites were in regions of intrinsic flexibility. The destabilization mechanism varied (Figure 3iii): At some sites, destabilization was entropic, reflecting increased conformational fluctuations, but enthalpic at other sites, reflecting weakened intramolecular interactions. In contrast, ubiquitination at Lys78 sites conferred a mild protective effect by transiently stabilizing a local structural motif through weak interactions between the C-terminal residues of ubiquitin and the substrate. Ubiquitination at destabilizing sites increased the population of partially unfolded, protease-accessible states, and the rate at which ubiquitinated substrates undergo degradation correlates with the degree of destabilization caused by ubiquitin modification. Interestingly, in constructs with unstructured initiation regions appended, monoubiquitination further accelerated degradation, suggesting ubiquitin can prime substrates even in the presence of other degron elements.

## FAT10 as a thermodynamic modulator of protein stability

Recent studies from our lab demonstrated that FAT10 destabilizes proteins in a site- and substrate-specific manner through conformational entropy and loss of enthalpic interactions, actively contributing to proteasomal engagement [23,24].

### FAT10’s intrinsic structural plasticity

We observed that FAT10 lacks long-range salt bridges and critical hydrophobic packing interactions, rendering it significantly less stable than ubiquitin. FAT10’s global unfolding free energy (ΔG_unfolding_ ≈ 2.3 kcal/mol) is markedly lower than that of ubiquitin (≈8 kcal/mol), with a melting temperature of 55°C compared with >95°C for ubiquitin. In this work [24], steered molecular dynamics and high-temperature Molecular dynamics (MD) simulations confirmed that FAT10 unfolds faster and with less mechanical work than ubiquitin, highlighting its ductile nature. Root mean square fluctuations in the backbone of FAT10, its hydrogen bonding patterns, and energy landscapes consistently indicate local flexibility at the β1-β2 loops and the α1 helices in both domains, rendering the protein partially unfolded. This local disorder results from the lack of essential stabilizing electrostatic interactions, such as the salt bridges observed in ubiquitin. As a result, FAT10 samples higher energy conformations more readily, facilitating its rapid mechanical unfolding and proteasomal degradation.

### Substrate destabilization via FAT10 conjugation

Cycloheximide chase assays in our lab demonstrated rapid turnover of FAT10-conjugated substrates, while the ubiquitin-conjugated substrates remained relatively stable [24]. We also demonstrated that FAT10 caused a dramatic decrease in the free energy of unfolding (ΔG_unfolding_) of substrates. The ΔG_unfolding_ of substrate Cellular retinoic acid-binding protein 1 (CRABP1) reduced from 5.9 kcal/mol in apo form to 2.4 kcal/mol when conjugated to fat10. In comparison, ubiquitin-conjugated CRABP1 had a modest 1.1 kcal/mol drop in ΔG_unfolding_. For ultra-stable proteins like Cyan fluorescent protein (CFP), we observed that FAT10 reduced stability 15-fold more than ubiquitin. This destabilization is attributed to non-specific but persistent intermolecular collisions that disrupt substrate contacts, creating proteasome-compatible partially unfolded conformations.

### Mechanistic determinants of FAT10-mediated destabilization

**Figure 4:**
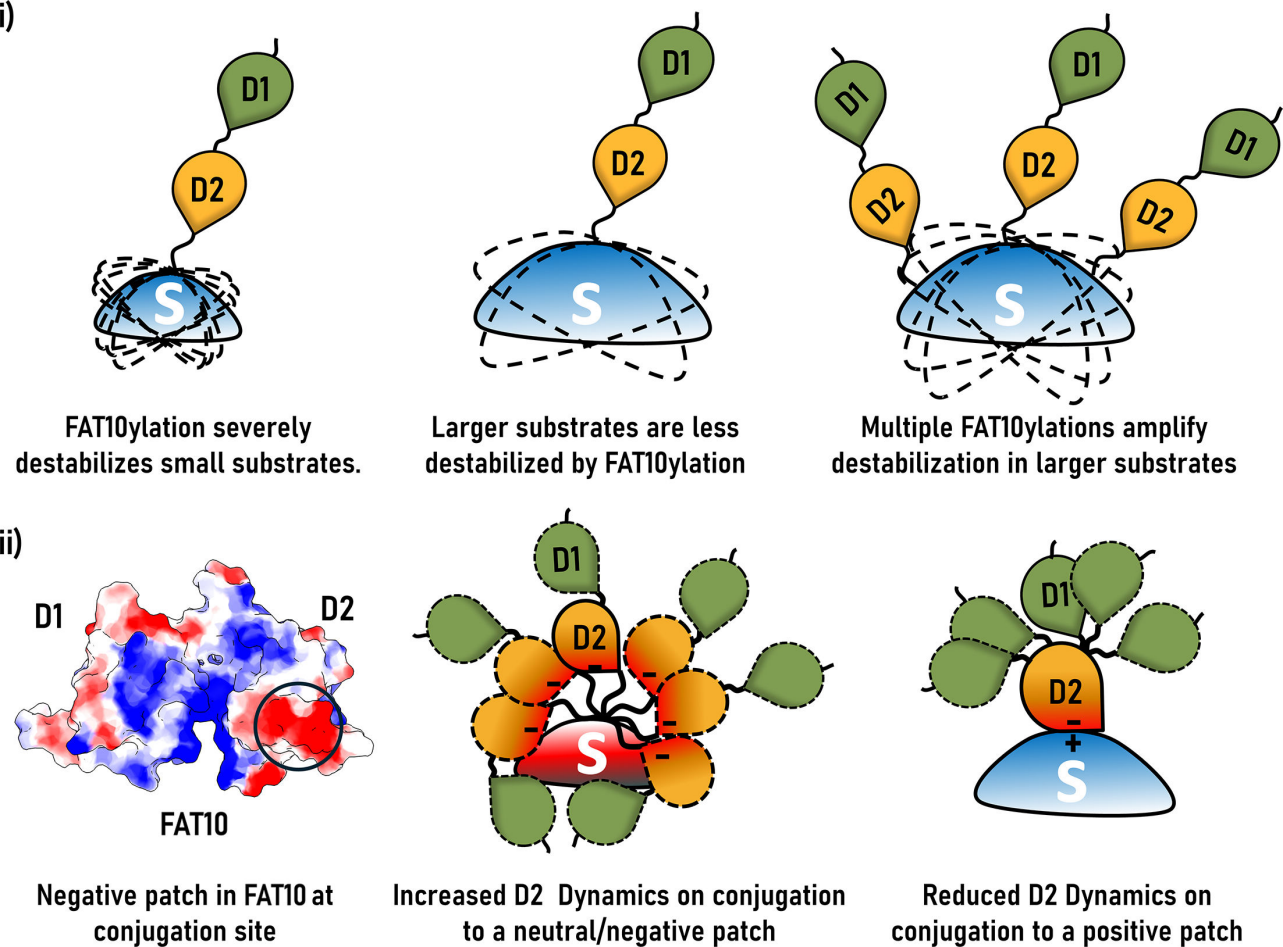
Thermodynamic determinants of FAT10-mediated destabilization. (**i**)Schematic illustrating the effect of substrate size and FAT10ylation stoichiometry on substrate destabilization. FAT10ylation severely destabilizes small substrates (left), while larger substrates are less affected (middle). However, multiple FAT10 moieties can synergistically amplify destabilization even in larger substrates (right). (**ii**) Electrostatic surface representation of FAT10 showing the distribution of surface charges (left), with negative regions in red and positive regions in blue. The D2 domain exhibits a prominent negative patch near the conjugation site. Conjugation of this negatively charged region to a neutral or positively charged substrate surface alters the dynamics of the D2 domain: increased flexibility is observed upon attachment to neutral surfaces/negative (middle), while conjugation to a positively charged patch restricts D2 dynamics (right).

Further MD simulations on multiple FAT10-conjugated substrates revealed distinct patterns of destabilization based on substrate properties [23]:

- Parkin-Ubl: A small, flexible substrate with a ubiquitin-like fold. FAT10ylation at three lysines significantly increased conformational entropy (Figure 4i). The effects of FAT10 conjugation were long range and could be observed in the distant regions of the substrate.
- Ube2z (USE1): A larger, compact substrate showed significant but lower destabilization than Parkin-Ubl (Figure 4i). FAT10 has a large negatively charged path near the conjugation site (Figure 4ii). Destabilization was greater when conjugation occurred at lysines located nearby negatively charged surface regions. Multi-mono-FAT10ylation at multiple sites further disrupted native contacts and increased entropy, highlighting two key principles: (i) Multivalency: multiple FAT10 tags amplify entropic penalties and disrupt long-range packing (Figure 4i). (ii) Surface electrostatics: conjugation of FAT10 to a positively charged patch in the substrate reduces electrostatic repulsion, promoting tighter interactions with FAT10 and enhancing destabilization.
- S15a: An intermediate size, positively charged surface showed moderate destabilization upon FAT10ylation (Figure 4ii). The entropy change was less than that observed in Ube2z, likely due to the restricted motion of FAT10’s D2 domain, caused by strong substrate–tag interactions.

## Conclusion

**Figure 5:**
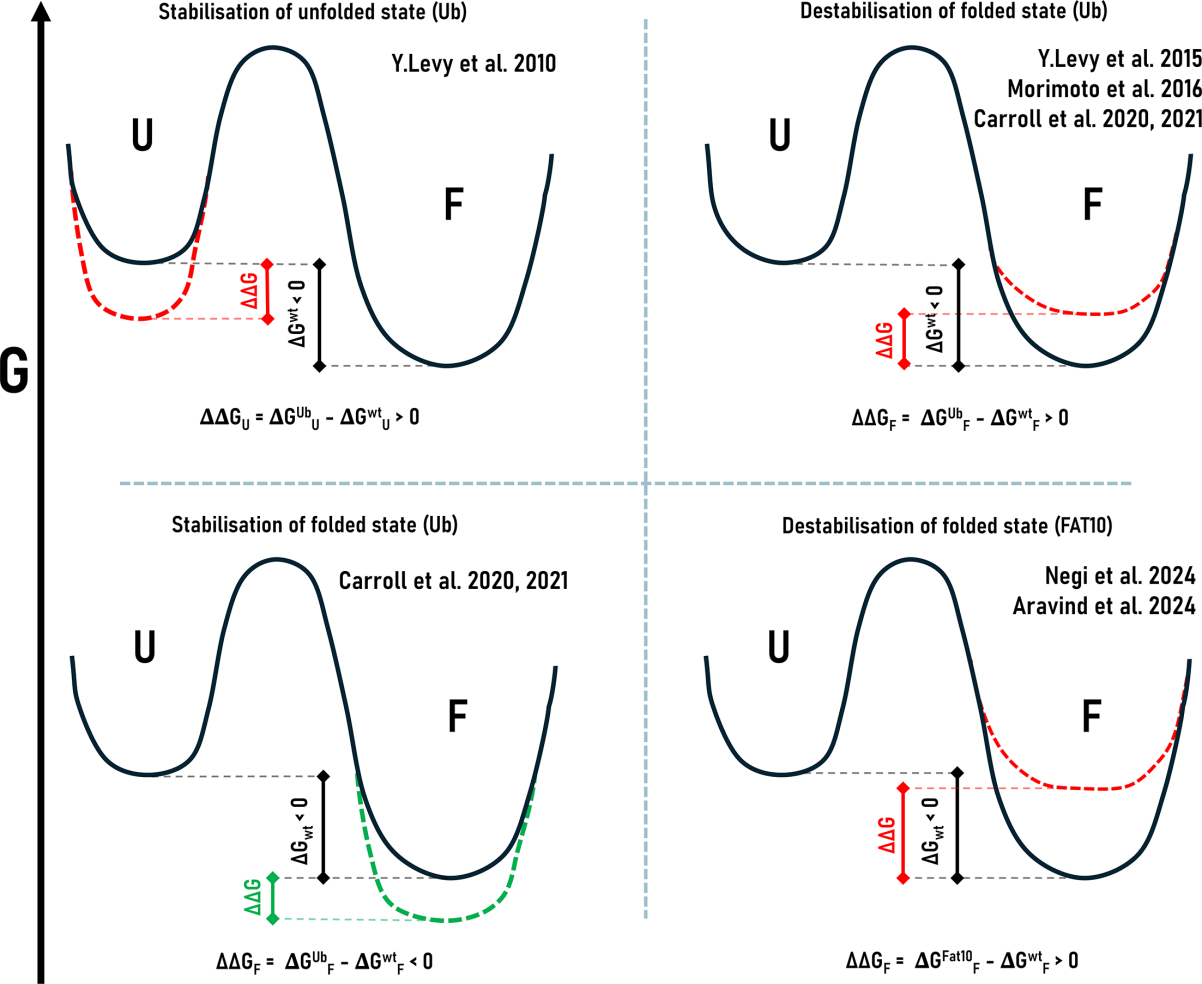
Modulation of free energy landscapes of tagged substrates as observed from various studies. Schematic representation of the folding free energy landscape of a substrate protein in its unmodified wild-type (black) and conjugated forms (colored dashed lines). The folded (**F**) and unfolded (**U**) states are separated by an energy barrier, with ΔG_F_ denoting the folding free energy. Upon conjugation, the energy difference changes to ΔG_conj_, and the resulting ΔΔG = ΔG_conj_ − ΔG_wt_ > 0 reflects net destabilization, or ΔΔG = ΔG_conj_ − ΔG_wt_ < 0 reflects net stabilization. Net stabilization of the folded state is represented by green dashed lines, whereas net destabilization is represented by red dashed lines. Destabilization of the unfolded state can result either from a decrease in entropy, which outweighs the enthalpic contribution. Alternatively, destabilization can arise from an increase in enthalpy, which is greater than the accompanying entropy gain. Net stabilization of the substrate is represented by green dashed lines, and net destabilization of the substrate upon conjugation is represented by red dashed lines. Four quadrants represent four different scenarios of substrate stabilization and destabilization observed in various studies.

While the degradation roles of ubiquitin and FAT10 are well established, comparative insights into how these tags influence substrate energy landscapes and mediate destabilization have only recently begun to emerge. Studies reveal [25–27,87–89] that ubiquitination can induce unstructured regions in otherwise folded proteins, circumventing the need for pre-existing disordered segments. Polyubiquitinated barstar variants lacking unstructured tails were degraded by the proteasome when ubiquitinated specifically at destabilizing sites, while ubiquitination at the stabilizing site resisted degradation. Hypothesized structural rationale encompasses substrate entropic change, entropic pulling forces at lysine induced by tag motion, and stabilizing intermediate conformational states through direct tag–substrate contacts. The data acquired from various techniques converge on the model where tag alters the energy landscape of substrates, favoring proteasome-engageable destabilized states (*ΔΔG = ΔG_conj_ − ΔG_wt_ >> 0*). Ubiquitin may restrict the conformational flexibility of the substrate, reducing the entropy (*ΔS_U_ = S_U_^conj^ − S_U_^wt^<0*) of the native state (Figure 5, top left), inducing entropically driven destabilization. Ubiquitination can also disrupt intra-substrate interactions, weakening hydrogen bonding networks, causing disorder in the substrate driven by enthalpy (*ΔH_U_ = H_U_^conj^ − H_U_^wt^>0*) (Figure 5, top right). At certain modification sites, ubiquitin had the opposite effect. Ubiquitin’s C-terminus forms non-covalent interactions to shield the substrate from destabilization (Figure 5, bottom left). This mirrors ubiquitin’s role in nondegradative processes, which may stabilize specific substrate conformations to facilitate downstream signaling. Notably, ubiquitin itself remained structurally stable during these interactions. This inherent stability may ensure ubiquitin retains the signaling integrity across diverse substrates. Collectively, these studies demonstrate that ubiquitination acts as more than a passive tag for degradation—it actively modulates substrate conformational landscapes in a site-dependent manner.

Compared with ubiquitin, the effect of FAT10 on the substrate stability is significantly higher (Figure 5, bottom right) with ∆∆G more than that of ubiquitin. FAT10’s low thermodynamic stability, lack of structural rigidity, and ability to induce conformational entropy in substrates accelerate proteasomal recognition and degradation. This effect is substrate-dependent and modulated by several factors, including the modification site, substrate size, intrinsic flexibility, and surface charge distribution. Smaller and inherently flexible substrates exhibit more pronounced destabilization, while larger, more rigid substrates like Ube2z require multi-mono-FAT10ylation to higher effects. Moreover, FAT10’s destabilizing influence is enhanced when conjugated to negatively charged or structured regions, which enhances its conformational mobility and nonspecific interactions. The effect on enthalpy is also elevated with significant loss of native contact in FAT10ylated substrates. Unlike ubiquitin, a unique feature of FAT10 is that it co-degrades with its substrate. This functional link is mirrored in its thermodynamic properties. FAT10 is more disordered when conjugated to substrates than in its free form [23,24], suggesting thermodynamic coupling to the substrate. It co-operatively increases the conformational fluctuations in the FAT10–substrate conjugate to increase disorder, making it prone to degradation at the proteasome.

The mechanistic insight obtained from studying ubiquitin–substrate and FAT10–substrate interactions has broad implications for these degradation pathways. First, it reveals a previously underappreciated layer of regulation in proteostasis—namely, site-specific modification can fine-tune degradation kinetics not only by signaling but also by physically reshaping substrate energy landscapes. It may be recalled that for RING and Ubox E3 ligases, the interactions of E2~Ub/E3/substrate complex determine the modification site. For HECT and RBR ligases, E3~Ub/substrate interactions determine the modification site. Minor dysregulation of interactions in these complexes may change the modification site and the substrate’s fate. Second, it suggests new strategies for designing therapeutics: by mimicking or blocking the destabilizing ubiquitination/FAT10ylation events, one could influence degradation pathways in diseases marked by aberrant proteostasis. Targeting FAT10-mediated degradation may be leveraged in inflammation, cancer, or neurodegeneration, where controlled destabilization of specific proteins could modulate disease outcomes. It is emerging that ubiquitin and F10, through nuanced modulation of substrate stability, are versatile regulators of their fate.

**Summary Points**

- Ubiquitin and FAT10 function beyond degradation tags, actively influencing substrate stability.
- Their effects are site- and context-dependent, altering the fate of tagged proteins.
- Ubiquitin modulates protein energetics via both entropic and enthalpic mechanisms, whereas FAT10 generally destabilizes substrates due to its structural flexibility and co-degradation mechanism.
- Observed biophysical consequences are critical for proteasomal recognition and processing. Insights from these findings pave the way for new strategies to modulate proteostasis.

## Open Access

This article has been published open access under our Subscribe to Open programme, made possible through the support of our subscribing institutions, learn more here: https://portlandpress.com/pages/open_access_options_and_prices#conditional

## Abbreviations

CP: core particle
HDX: hydrogen-deuterium exchange
MHC: major histocompatibility complex
RP: regulatory particle
VCP: valosin-containing protein
